# Validation of image segmentation by estimating rater bias and variance

**DOI:** 10.1098/rsta.2008.0040

**Published:** 2008-04-11

**Authors:** Simon K. Warfield, Kelly H. Zou, William M. Wells

**Affiliations:** 1 Computational Radiology Laboratory, Department of Radiology, Children's Hospital, Harvard Medical School300 Longwood Avenue, Boston, MA 02115, USA; 2 Department of Radiology, Brigham and Women's Hospital, Harvard Medical School221 Longwood Avenue, Boston, MA 02115, USA

**Keywords:** segmentation, validation, expectation-maximization, magnetic resonance imaging, MRI, tumour

## Abstract

The accuracy and precision of segmentations of medical images has been difficult to quantify in the absence of a ‘ground truth’ or reference standard segmentation for clinical data. Although physical or digital phantoms can help by providing a reference standard, they do not allow the reproduction of the full range of imaging and anatomical characteristics observed in clinical data.

An alternative assessment approach is to compare with segmentations generated by domain experts. Segmentations may be generated by raters who are trained experts or by automated image analysis algorithms. Typically, these segmentations differ due to intra-rater and inter-rater variability. The most appropriate way to compare such segmentations has been unclear.

We present here a new algorithm to enable the estimation of performance characteristics, and a true labelling, from observations of segmentations of imaging data where segmentation labels may be ordered or continuous measures. This approach may be used with, among others, surface, distance transform or level-set representations of segmentations, and can be used to assess whether or not a rater consistently overestimates or underestimates the position of a boundary.

## 1. Introduction

The segmentation of structures from medical images is a key objective in many quantitative and qualitative applications of imaging. A variety of different segmentation approaches have been developed and exploited different characteristics of images. To evaluate the success of a particular segmentation approach, it is common to compare with segmentations obtained interactively by experts. When experts outline structures interactively, they may infer the location of the boundary of a structure from the imaging data.

Validation may be carried out by comparison with either the boundary or the region enclosed by the boundary. Similarly, some segmentation algorithms use a boundary representation directly, whereas others use regions or local neighbourhood information.

Previous work for estimating a reference standard from segmentations has used the expectation-maximization (EM) algorithm to estimate performance characteristics and the hidden ‘true’ segmentation from a collection of independent binary segmentations, indicating the presence or absence of a structure in a given image (Warfield *et al*. 2002), and from a collection of segmentations with multi-category labellings, such as grey matter, white matter and cerebrospinal fluid (Warfield *et al*. 2004). The method has been used to characterize image segmentations (Rohlfing *et al*. 2003) and to infer labellings from repeated registrations (Rohlfing *et al*. 2004). These approaches are not appropriate when an ordering is present in the representation of the segmentation, such as when the segmentation boundary is represented by a signed distance transform or level set, and, in other representations, where a continuous score is present at each voxel.

When an expert or algorithm outlines a boundary, there may be a systematic bias in the placement of the boundary, and we would like to be able to characterize and compensate for such a bias. In this paper, we present an algorithm, first reported in Warfield *et al*. (2006), which aims to characterize the performance of segmentation generators where the segmentation is represented by a boundary, and key performance parameters for a particular segmentation relate to the potential bias and variance in the boundary placement. In this paper, we have carried out an updated and expanded evaluation of the algorithm of Warfield *et al*. (2006).

The objective of this paper was to generalize the existing methodology in simultaneous ground truth estimation and performance level estimation, as described in Warfield *et al*. (2004), to segmentations represented by a continuous score per voxel. Here we propose a model in which we summarize the quality of a rater-generated segmentation by the mean and variance of the distance between the segmentation boundary and a reference standard boundary. We propose an EM algorithm to estimate the reference standard boundary and the bias and variance of each rater. This enables interpretation of the quality of a segmentation generator, be it a human expert or an algorithm. A good quality rater will have a small average distance from the true boundary (low bias) and a high precision (small variance of distance from the true boundary).

We applied the proposed algorithm to the assessment of brain tumour segmentations. We evaluated the algorithm by testing its operation on phantoms to confirm its operation with a known reference standard, and we assessed the significance of the method by comparing and contrasting with other means of measuring segmentation similarities using the Dice (1945) overlap measure and STAPLE (Warfield *et al*. 2004).

## 2. Method

### (a) Notation and assumptions

We observe a set of segmentations of an image, created by some number of human expert or algorithmic raters. It is our goal to estimate the true labelling of the image and to estimate the performance characteristics of each rater in comparison. The true labelling is unknown (hidden), but if it was not hidden, then it would be straightforward to compute the bias and variance parameters for each rater, characterizing the way in which the rater labelling differs from the true labelling. Since the true labelling is hidden, here we describe an EM algorithm (Dempster *et al*. 1977) in order to estimate the true labelling and rater performance parameters. Our EM algorithm proceeds iteratively, first estimating the complete data log-likelihood function, and then identifying the parameters that maximize the estimated complete data log-likelihood function. The estimation of the complete data log-likelihood involves computing the expected value of this function conditioned upon the observed rater labellings and previous estimates of the rater parameters. Computation of this expectation requires the estimation of the conditional probability of the true score given the observed scores and rater performance parameters. The iteration procedure is guaranteed to converge locally to the maximum of the observed data likelihood.

Let *i*=1, …, *I* index the raters generating a segmentation of an image and *j*=1, …, *J* index the voxels in the image. The label of each voxel is a continuous scalar, such as may be obtained from a distance transform or level-set representation of a segmentation (but is not restricted to those sources) and is referred to as a score. The score *s*_*ij*_ assigned by rater *i* for voxel *j* has the following composition:

(2.1) $$s_{ij}=\tau_{j}+\beta_{i}+\epsilon_{ij},$$

where *τ*_*j*_ is the underlying true score for this voxel and *β*_*i*_ is the bias of rater *i*. The error term is assumed to have an uncorrelated normal distribution, i.e. $\epsilon_{ij}∼N(0,\sigma_{i}^{2})$, for each rater *i*. That is, we characterize the rater performance by a bias *β*_*i*_ and a variance $\sigma_{i}^{2}$. We assume that, given an image to be scored, the raters score the image independently.

We wish to estimate the bias and variance that characterize the performance of each rater. If the true score was known, we could estimate the rater parameters *θ*=(*σ*, *β*) by solving the *complete data* log-likelihood problem,

(2.2) $$\hat{\theta }=\text{arg}\;\underset{\theta }{\text{max}}\;\text{log}\;Pr(s,\tau |\sigma ,\beta ).$$

Since the true score is unknown, we instead estimate the complete data log-likelihood using the EM algorithm (McLachlan & Krishnan 1996) by computing its expected value under a conditional probability density function (pdf) for the true scores given the rater scores and previous estimates of the rater parameters. That is, we identify the rater parameters by solving

(2.3) $$\theta^{\left(t\right)}=\text{arg}\;\underset{\theta }{\text{max}}\;E\left[\text{log}\;Pr(s,\tau |\sigma ,\beta )|s,\theta^{\left(t-1\right)}\right],$$

where the conditional expectation is evaluated with respect to $p(\tau |s,\theta^{\left(t-1\right)})$. In order to compute this expectation, we need to evaluate the conditional probability density for the true scores given the rater scores and rater parameter estimates. We now derive the required densities and estimators.

In the absence of spatial correlations between voxels, the joint distribution of the scores of all the voxels conditional upon the true scores and rater parameters is assumed to have the form

(2.4) $$Pr(s|\tau ,\sigma ,\beta )=\prod_{j=1}^{J}\prod_{i=1}^{I}\phi \left\{\frac{s_{ij}-(\tau_{j}+\beta_{i})}{\sigma_{i}}\right\},$$

where *ϕ*{·} is the pdf of the standard normal distribution, *N*(0, 1). For notational simplicity, we write the pdf *N*(*μ*, *σ*^2^) as *ϕ*_*σ*_(*μ*).

Equation (2.4) states the key assumption of our approach. Restating this assumption, we assume that each rater is independently assigning scores, and that the error in assigning a score, after accounting for the rater-specific bias and rater-specific variance, has the standard normal distribution. Here we have also assumed that there is no spatial correlation among true scores, but this assumption could be easily relaxed by incorporating a Markov random field model for the spatial dependence of the true scores, as has been used in other related work (Warfield *et al*. 2004).

### (b) A conditional pdf for the true scores

In this section, we apply Bayes' theorem in order to derive the posterior distribution Pr(τ|s,σ,β) from the distribution of the observed scores Pr(s|τ,σ,β).

Since the true score is independent of the rater bias and variance, the posterior distribution is

(2.5) $$Pr(\tau |s,\sigma ,\beta )=Pr(s|\tau ,\sigma ,\beta )\cdot \frac{Pr(\tau ,\sigma ,\beta )}{Pr(s,\sigma ,\beta )},$$

(2.6) $$Pr(\tau |s,\sigma ,\beta )=Pr(s|\tau ,\sigma ,\beta )\cdot \frac{Pr(\tau )Pr(\sigma ,\beta )}{Pr(s,\sigma ,\beta )},$$

(2.7) $$Pr(\tau |s,\sigma ,\beta )=Pr(s|\tau ,\sigma ,\beta )\cdot \frac{Pr(\tau )}{Pr(s|\sigma ,\beta )}.$$

Upon integrating the expression of equation (2.7) over *τ*, since the marginal distribution integrates to 1, we have

(2.8) $$\int_{\tau }Pr(\tau |s,\sigma ,\beta )\;\text{d}\tau =\int_{\tau }Pr(s|\tau ,\sigma ,\beta )\cdot \frac{Pr(\tau )}{Pr(s|\sigma ,\beta )}\;\text{d}\tau ,$$

(2.9) $$1=\frac{1}{Pr(s|\sigma ,\beta )}\int_{\tau }Pr(s|\tau ,\sigma ,\beta )Pr(\tau )\;\text{d}\tau ,$$

and on substituting the expression from equation (2.7) for Pr(s|σ,β), we have

(2.10) $$Pr(\tau |s,\sigma ,\beta )=\frac{Pr(s|\tau ,\sigma ,\beta )Pr(\tau )}{\int_{\tau }Pr(s|\tau ,\sigma ,\beta )Pr(\tau )\;\text{d}\tau }.$$

We have considerable freedom in choosing the prior distribution on *τ*,

(2.11) $$Pr(\tau )=\prod_{j=1}^{J}Pr(\tau_{j}).$$

A multivariate Gaussian distribution is a natural choice, and in the absence of other information we may choose a multivariate uniform distribution, with scale parameter *h*,

(2.12) $$Pr(\tau )=\prod_{j=1}^{J}U_{h}(\tau_{j})=\frac{1}{h^{J}},$$

which simplifies the form of the posterior:

(2.13) $$Pr(\tau |s,\sigma ,\beta )=\frac{Pr(s|\tau ,\sigma ,\beta )\left(1/h^{J}\right)}{\left(1/h^{J}\right)\int_{\tau }Pr(s|\tau ,\sigma ,\beta )\;\text{d}\tau },$$

(2.14) $$Pr(\tau |s,\sigma ,\beta )=\frac{Pr(s|\tau ,\sigma ,\beta )}{\int_{\tau }Pr(s|\tau ,\sigma ,\beta )\;\text{d}\tau }.$$

Let the bias-adjusted score be denoted by $\mu_{ij}=s_{ij}-\beta_{i}$. From equations (2.4) and (2.11), we have

(2.15) $$Pr(\tau |\sigma ,\mu )=\frac{1}{Z}\prod_{j=1}^{J}Pr(\tau_{j})\prod_{i=1}^{I}\phi_{\sigma_{i}}(\mu_{ij}-\tau_{j}),$$

where the normalizing constant is

(2.16) $$Z=\int_{\tau_{J}}\ldots \int_{\tau_{1}}\prod_{j=1}^{J}Pr(\tau_{j})\prod_{i=1}^{I}\phi_{\sigma_{i}}(\mu_{ij}-\tau_{j})\;\text{d}\tau_{1}\ldots \;\text{d}\tau_{J}.$$

Therefore, we can write for each voxel,

(2.17) $$Pr(\tau_{j}|\mu ,\sigma )=\frac{1}{Z_{j}}Pr(\tau_{j})\prod_{i=1}^{I}\phi_{\sigma_{i}}(\mu_{ij}-\tau_{j})=\frac{1}{Z_{j}}Pr(\tau_{j})\prod_{i=1}^{I}\frac{1}{\sqrt{2\pi }\sigma_{i}}\;\text{exp}\;\left\{-\frac{{\left(\mu_{ij}-\tau_{j}\right)}^{2}}{2\sigma_{i}^{2}}\right\}=\frac{1}{Z_{j}}\left\{\prod_{i=1}^{I}{\left(2\pi \sigma_{i}^{2}\right)}^{-1/2}\right\}\;\text{exp}\;(w_{ij})Pr(\tau_{j}),$$

which is a product of normal distributions with mean *μ*_*ij*_ and variance $\sigma_{i}^{2}$, and where

(2.18) $$w_{ij}=-\frac{1}{2}\sum_{i=1}^{I}\frac{{\left(\mu_{ij}-\tau_{j}\right)}^{2}}{\sigma_{i}^{2}}=-\frac{1}{2}\sum_{i=1}^{I}\frac{1}{\sigma_{i}^{2}}(\mu_{ij}^{2}-2\mu_{ij}\tau_{j}+\tau_{j}^{2})=-\frac{1}{2}\left[\sum_{i=1}^{I}\frac{\mu_{ij}^{2}}{\sigma_{i}^{2}}-2\tau_{j}\sum_{i=1}^{I}\frac{\mu_{ij}}{\sigma_{i}^{2}}+\tau_{j}^{2}\sum_{i=1}^{I}\frac{1}{\sigma_{i}^{2}}\right].$$

If *Pr*(*τ*_*j*_) of equation (2.17) is uniform, then completing the square and identifying the terms in a Gaussian pdf yields the following variance and mean terms:

(2.19) $$\frac{1}{\sigma^{2}}=\sum_{i=1}^{I}\frac{1}{\sigma_{i}^{2}},$$

(2.20) $$\mu_{j}=\frac{\sum_{i=1}^{I}\left(s_{ij}-\beta_{i}\right)/\sigma_{i}^{2}}{1/\sigma^{2}}.$$

If *Pr*(*τ*_*j*_) of equation (2.17) is distributed as $N(\mu_{\tau_{j}},\sigma_{\tau_{j}}^{2})$, then it acts analogously to another rater with the specified mean and variance.

Observe that the mean score for voxel *j*, *μ*_*j*_, indicated by this distribution is an inverse rater variance-weighted sum of the difference between the score of the rater and the rater bias, and that the variance of the distribution is the harmonic mean of the rater variances.

### (c) Estimating the complete data log-likelihood

The parameters maximizing the conditional expectation of the complete data log-likelihood can be found from

(2.21) $$\theta^{\left(t\right)}=\text{arg}\;\underset{\theta }{\text{max}}\;E\left[\text{log}\;Pr(s,\tau |\sigma ,\beta )|s,\theta^{\left(t-1\right)}\right],$$

(2.22) $$\theta^{\left(t\right)}=\text{arg}\;\underset{\theta }{\text{max}}\;E\left[\text{log}\;Pr(s|\tau ,\sigma ,\beta )|s,\theta^{\left(t-1\right)}\right],$$

(2.23) $$\theta^{\left(t\right)}=\text{arg}\;\underset{\sigma ,\beta }{\text{max}}\;E\left[\sum_{i=1}^{I}\sum_{j=1}^{J}\text{log}\left(\frac{1}{\sqrt{(}2\pi \sigma_{i}^{2})}\;\text{exp}\;\left(-\frac{1}{2\sigma_{i}^{2}}{\left(\mu_{ij}-\tau_{j}\right)}^{2}\right)\right)|s,\theta^{\left(t-1\right)}\right],$$

(2.24) $$\theta^{\left(t\right)}=\text{arg}\;\underset{\sigma ,\beta }{\text{max}}\;\sum_{i=1}^{I}\sum_{j=1}^{J}\left[-\text{log}\;\sigma_{i}-\frac{1}{2\sigma_{i}^{2}}\left(\mu_{ij}^{2}-2\mu_{ij}E(\tau_{j})+E\left(\tau_{j}^{2}\right)\right)\right].$$

Now, given the distribution Pr(τ|s,σ,β) above, we have

(2.25) $$E(\tau_{j})=\mu_{j}^{\left(t-1\right)},$$

(2.26) $$E(\tau_{j}^{2})=\text{var}(\tau_{j})+E{\left(\tau_{j}\right)}^{2},$$

(2.27) $$E(\tau_{j}^{2})={\left(\sigma^{2}\right)}^{\left(t-1\right)}+{\left(\mu_{j}^{2}\right)}^{\left(t-1\right)}.$$

Hence,

(2.28) $$\theta^{\left(t\right)}=\text{arg}\;\underset{\sigma ,\beta }{\text{max}}\;\sum_{i=1}^{I}\sum_{j=1}^{J}\left[-\text{log}\;\sigma_{i}-\frac{1}{2\sigma_{i}^{2}}\left(\mu_{ij}^{2}-2\mu_{ij}\mu_{j}^{\left(t-1\right)}+{\left(\sigma^{2}\right)}^{\left(t-1\right)}+{\left(\mu_{j}^{2}\right)}^{\left(t-1\right)}\right)\right].$$

On differentiating equation (2.28) with respect to the parameters *β* and *σ* and solving for a maximum, we find the following estimators for the rater performance parameters:

(2.29) $$\beta_{i}^{\left(t\right)}=\frac{1}{J}\sum_{j=1}^{J}\left(s_{ij}-\tau_{j}^{\left(t-1\right)}\right),$$

(2.30) $${\left(\sigma_{i}^{2}\right)}^{\left(t\right)}=\frac{1}{J}\sum_{j=1}^{J}{\left(s_{ij}-\beta_{i}^{\left(t\right)}-\tau_{j}^{\left(t-1\right)}\right)}^{2}+{\left(\sigma^{2}\right)}^{\left(t-1\right)}.$$

The rater bias estimator is the average observed difference between the rater score and the previously estimated true score over all the voxels, and the rater variance estimator is a sum of a term describing a natural empirical variance estimator (given the estimates of the rater bias and the true score) and a term that is the harmonic mean of the previous estimates of the rater variances over all the raters. Thus, the estimated variance for a rater cannot be smaller than the variance associated with the true score estimate.

## 3. Results

We applied the proposed estimation scheme to a set of digital phantoms, generated by synthetic raters with pre-specified bias and variance parameters, in order to determine whether the estimation scheme would correctly identify the bias and variance of segmentation generators for which we knew the true parameter values.

We applied the proposed estimation scheme to MRI scans of four different brain tumours. We compared the estimated true contour with that obtained from averaging the segmentations. We compared the segmentations identified as best and worst by the algorithm with the original MRI scan visually, and by computing a spatial overlap measure between the reference standard and each segmentation.

### (a) Estimation of parameters of synthetic raters using a digital phantom

**Figure 1:**
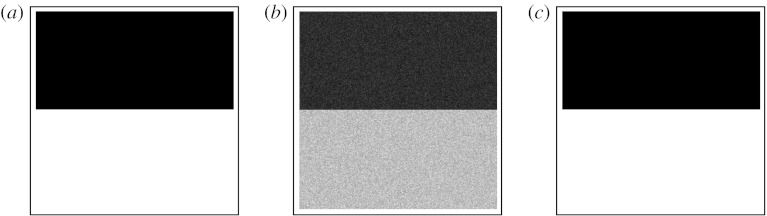
Estimated true labels from synthetic raters. The specified labelled data were created with two regions of intensity equal to 100 and 200, respectively. Five raters with a bias of +10 units and a variance of 100 units and five raters with a bias of −10 units and a variance of 50 units were simulated to create synthetic segmentations. The estimation scheme was used to estimate each rater bias and variance, and the hidden true labelling. As can be seen in (*c*), despite the noisy and limited observations such as that shown in (*b*), the estimated reference standard appears very similar to the specified data of (*a*). The closeness of the estimated parameters for each of the raters to the specified values confirms that the estimate is effective. (*a*) Synthetic true labelling. (*b*) Rater segmentation with a bias of +10 units and a variance of 100 units. (*c*) Estimated true labelling.

Segmentations were generated by randomly perturbing an image consisting of two rectangular regions of intensity equal to 100 and 200, respectively. Ten random segmentations were generated, drawing from five synthetic raters with a bias of +10 units and a variance of 100 units and five synthetic raters with a bias of −10 units and a variance of 50 units. The estimation scheme was executed and estimates of the true image and of the performance characteristics of each rater were obtained. This is illustrated in figure 1. The estimated bias was 9.9968±0.003 and −9.9968±0.0002, which is a tight estimate around the true value. The estimated variance was 100.16±0.28 and 50.112±0.101, which again is very close to the specified values of the rater variances.

### (b) Segmentations of MRI of brain tumours by human raters

**Figure 2:**
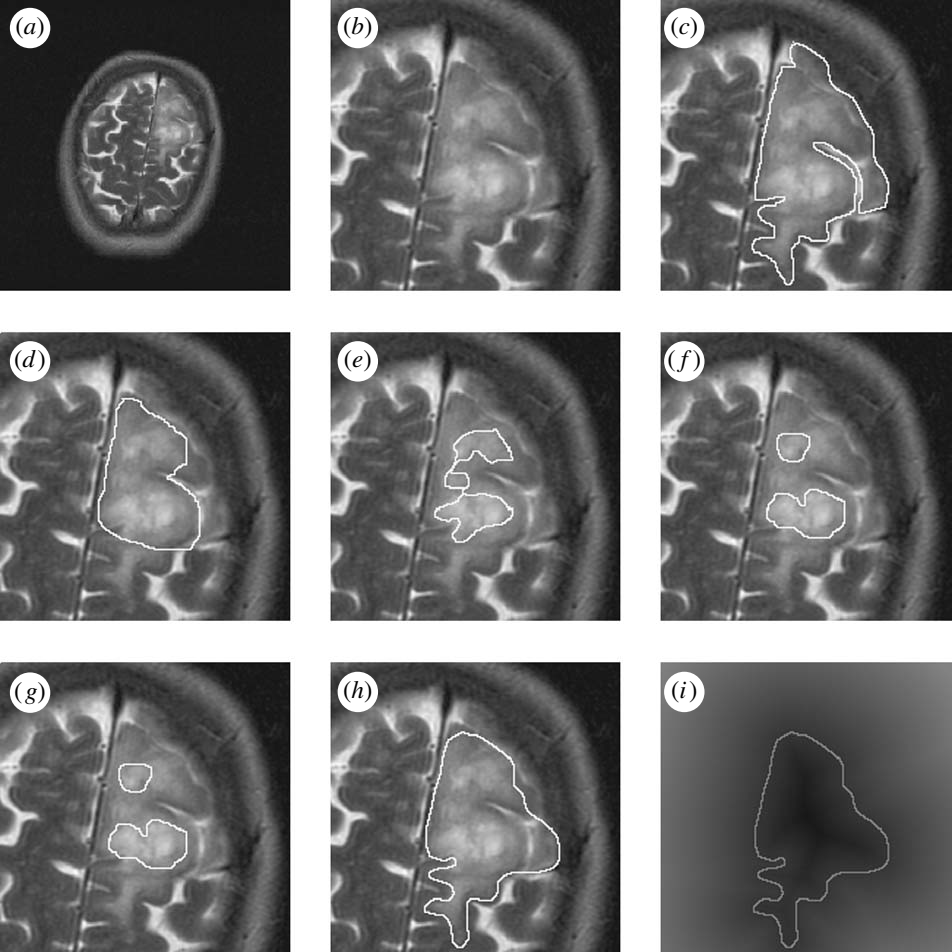
Illustration of MRI showing a brain tumour, and a set of boundaries delineated by different raters. Each boundary was drawn interactively by the rater to delineate the extent of the tumour that the rater perceived. A signed distance transform of each contour is calculated, with the voxels inside the contour at negative distances from the boundary and the voxels outside the contour at positive distances from the boundary. (*a*) MRI of a brain tumour. (*b*) Cropped region of interest. (*c*) Rater 1 contour. (*d*) Rater 2 contour. (*e*) Rater 3 contour. (*f*) Rater 4 contour. (*g*) Rater 5 contour. (*h*) Rater 6 contour. (*i*) Signed distance transform of rater 6 contour.

Each brain tumour was segmented up to three times by each of nine raters for a maximum of 27 segmentations. Each rater segmentation consisted of a closed contour delineating the extent of the tumour that the rater perceived in the MRI. A signed distance transform of each contour was computed in order to obtain a segmentation with each voxel representing the shortest distance from the boundary. An illustration of one MRI of a brain tumour with corresponding boundaries delineated by the rater is shown in figure 2.

**Figure 3:**
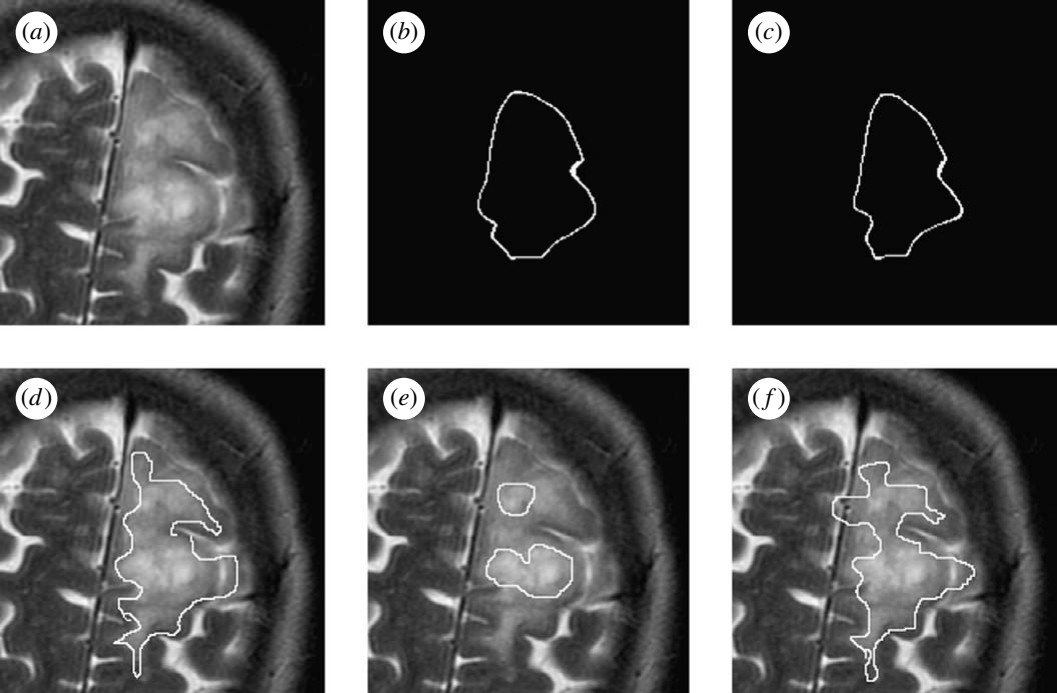
Estimated true segmentation from human rater segmentations. The estimated true contour is consistent with the human raters, and has a better spatial overlap with the rater segmentations, as measured by the Dice coefficient, than with the average segmentation. The best, worst and median rater segmentations as determined by the algorithm are also displayed. (*a*) MRI of a brain tumour. (*b*) Average contour. (*c*) Estimated true contour. (*d*) Best rater segmentation. (*e*) Worst rater segmentation. (*f*) Median rater segmentation.

The estimation scheme was executed to find the best overall reference standard using all of the segmentations. The scheme was run for 100 iterations, resulting in the sum of total estimated true scores changing by less than 0.01 at the final iteration, which required less than 90 s of computation time on a PowerBook G4 in each case. One illustrative brain tumour is shown in figure 3, together with the average contour and the estimated true contour. In addition, the rater segmentations were evaluated based on the bias identified by the algorithm, and the rater segmentations were ranked based on the bias. Shown are the rater segmentations that are most alike, most different from and the median segmentation from the true contour, on the basis of the rater bias.

We sought to summarize the spatial overlap between the rater segmentations, the average segmentation enclosed by the contour obtained by averaging each of the signed distance transforms and the segmentation defined by the zero level set of the estimation procedure. Different raters perceived the tumour boundary differently, and we measured the pairwise spatial overlap using the Dice measure. The coefficient of variation of the Dice overlap measures between the estimated reference segmentation and each of the rater segmentations was 0.15, which was smaller than the coefficient of variation of the Dice overlap measures between the average segmentation and the raters (0.17) and that between each rater and the other raters (which ranged from the smallest of 0.16–0.36). This indicates that there was considerable variability between the raters, but that the estimated segmentation was more consistent with the raters than with both the average of the segmentations and the other rater segmentations, and so is a good summary of the segmentations.

**Figure 4:**
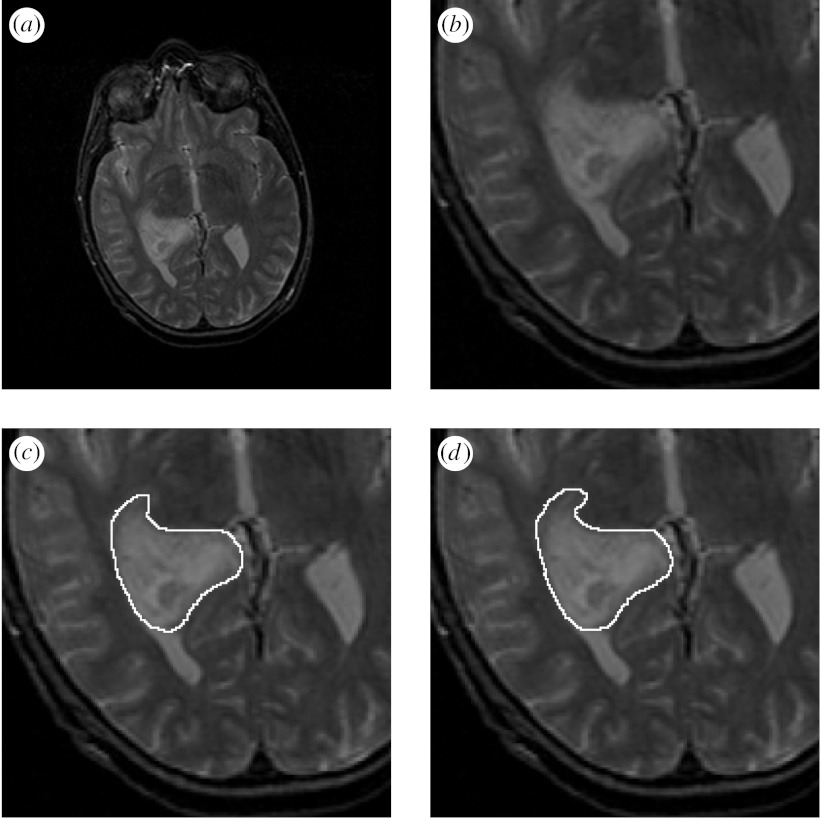
Illustration of reference segmentation estimation with a tumour near the lateral ventricles. Average and estimated true tumour contours from human rater segmentations, binarized by thresholding the estimated true score at 0.0. (*a*) MRI of a brain tumour. (*b*) Region of interest. (*c*) Contour from the average of rater distance transforms. (*d*) Estimated reference standard contour.

**Figure 5:**
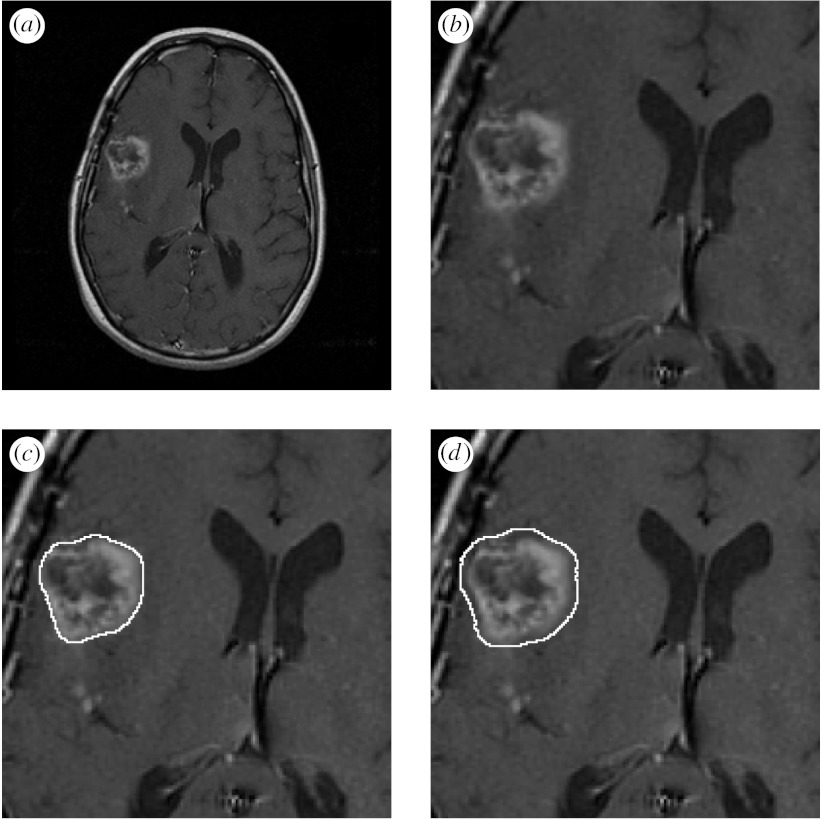
Average and estimated true tumour segmentations from human rater segmentations, binarized by thresholding the estimated true score at 0.0. (*a*) MRI of a brain tumour. (*b*) Region of interest. (*c*) Average of rater segmentations. (*d*) Estimated tumour segmentation.

**Figure 6:**
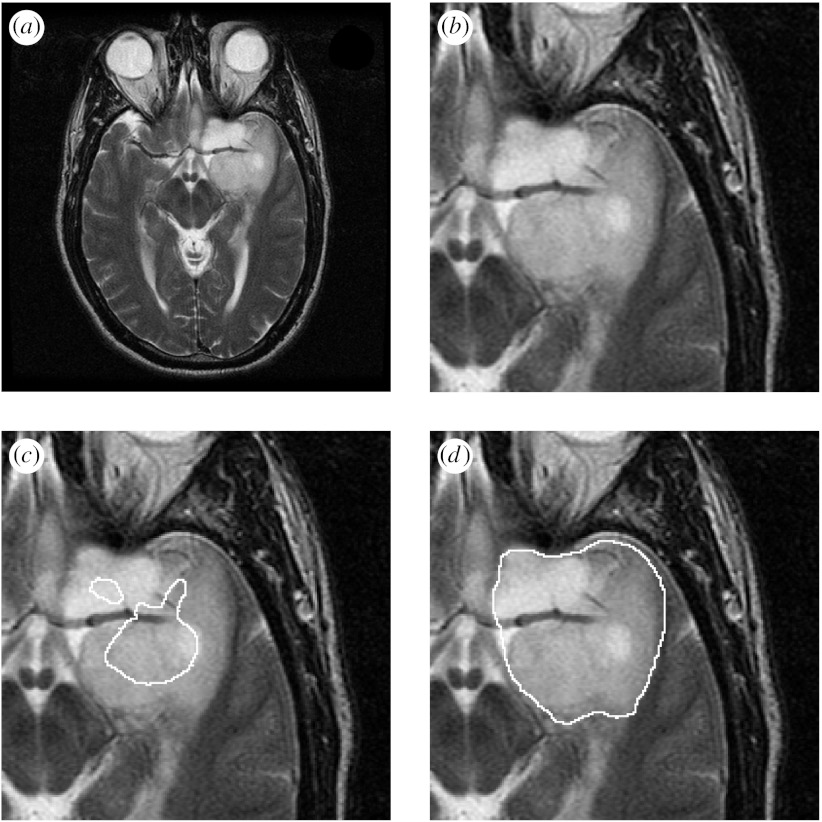
Average and estimated true tumour segmentations from human rater segmentations, binarized by thresholding the estimated true score at 0.0. (*a*) MRI of a brain tumour. (*b*) Region of interest. (*c*) Binarized average of rater segmentations. (*d*) Binarized estimated tumour segmentation.

Figures 4–6 illustrate the estimation procedure applied to three different tumours. When the tumour has a high boundary contrast and is easily recognized, the average is similar to that obtained with the estimation procedure accounting for the rater bias and variance, but when the tumour has a boundary that is challenging to identify, the average is not similar to that obtained with the estimation procedure. For boundaries that are difficult to identify, the differences in individual rater performance are important to take into account, and our proposed estimation scheme enables the estimation of the tumour segmentation that corresponds visually with the extent of the tumour perceived in the MRI scan.

### (c) Comparison with reference standard estimation from binary segmentations

Although the identification of a boundary is the critical task for segmentations in this setting, we can create a binary segmentation from the boundary by identifying the regions inside and outside the boundary. This then allows us to apply spatial overlap measures and previously described approaches to estimating a reference standard segmentation from binary segmentations, such as STAPLE (Warfield *et al*. 2004).

We binarized the rater segmentations and used STAPLE to make an estimate of the reference standard segmentation, and to estimate the sensitivity, specificity, positive predictive value and negative predictive value of each rater segmentation. We binarized the estimated reference standard segmentation at 50% probability, and computed spatial overlap measures between the binarized segmentations and the binarized reference standard.

**Figure 7:**
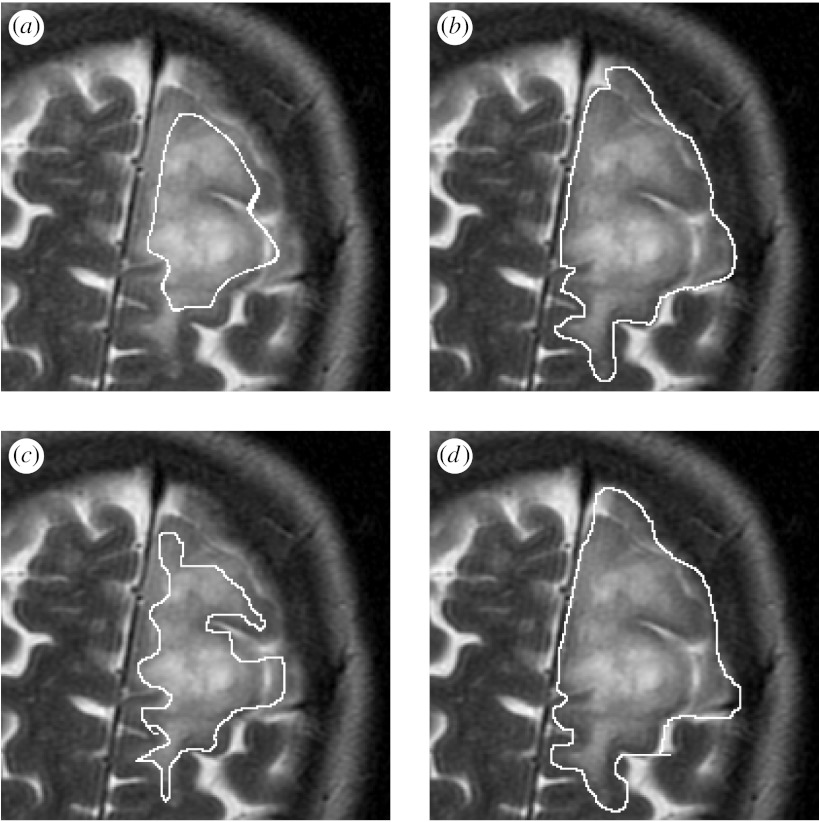
Comparison of the new algorithm and the binary STAPLE for the estimation of reference standard segmentation. (*a*) Overlay of contour estimate. (*b*) Overlay of STAPLE reference estimate. (*c*) Best rater segmentation identified with bias. (*d*) Best rater segmentation identified with sensitivity.

Figure 7 illustrates the reference standard segmentations estimated using the algorithm proposed here, the best segmentation identified by this algorithm as having the smallest rater bias, the binarized reference standard from STAPLE and the best segmentation identified by STAPLE as having the highest sensitivity.

The best contour estimate computed using the distance transform representation, illustrated in figure 7, appears to underestimate the tumour boundary, as it does not follow some of the intricate shape changes of the boundary. However, both approaches identify the same rater segmentations as the worst segmentations, most distant from the rater segmentations. Figure 7 also illustrates the segmentations identified as best by each algorithm. The Dice coefficient of spatial overlap between the reference standard estimates and the best segmentations identified with each method is reported in table 1.

## 4. Discussion and conclusion

Validation of image segmentations using an estimated reference standard is a valuable strategy for clinical imaging data where the true segmentation is unknown. Previous algorithms were designed for binary or unordered multi-category labels. Such methods may strongly penalize segmentations that may differ by small mislocalizations, and may not be appropriate for boundary- or surface-based segmentation algorithms.

The novel approach developed here is suitable for segmentations represented by a surface or boundary from which a distance may be computed, or for boundaries represented directly by a level set. This may be especially well suited to complicated structures where the true boundary is challenging to estimate, such as in the MRI of brain tumours, or where spatial mislocalization of the segmentation is expected and should be tolerated. An example of the latter situation would be in the estimation of centre lines of blood vessels, or the colon, or in the analysis of spicules in mammography.

We demonstrated that the estimation scheme was able to recover the true parameters of synthetic raters, and from this form a good estimate of the true image structure using digital phantoms. We demonstrated that, from a collection of human segmentations of brain tumours, the estimation scheme was able to identify a reference standard that was closer to the rater segmentations than the raters were to each other. We compared the reference standard with an estimate obtained by averaging and the results demonstrate that the average segmentation is less representative of the true anatomy. We demonstrated that the estimation scheme was able to rank the human raters, identifying the best and worst segmentations and providing valuable estimates of the rater bias and variance.

Future work will examine the possibility of incorporating a model for spatial correlation in the true scores, which will enable us to relax the assumption of voxelwise independence. It will also be interesting to examine alternatives for the prior probability of each score, such as may be derived from an anatomical atlas.

It would be interesting to assess the value of incorporating a model of spatial dependence of the true scores, such as by introducing a Markov random field model for the spatial dependence of the true scores. In an earlier work (Warfield *et al*. 2004) with unordered segmentation labels, we have found such a model helpful when the segmentations are particularly noisy, but, in the more common case of practical segmentations exhibiting high spatial dependence, a prior model that favours spatially correlated reference standard labellings can be valuable but is not critically important.

It would also be of interest to use local rather than global rater bias and variance parameters. If the rater bias and variance are allowed to vary across the image, it may be possible to identify regions in the image which are particularly challenging to segment, and may better model a rater that sometimes has a positive and sometimes a negative bias depending on the local image features.

## Figures and Tables

**Table 1:** Comparison of Dice coefficient overlap.

Dice coefficients	STAPLE standard	contour standard	contour best segmentation
STAPLE standard	1.0	0.67	0.72
contour standard	0.67	1.0	0.81
STAPLE best segmentation	0.94	0.63	0.68

## References

[bib1] Dempster A., Laird N., Rubin D. (1977). Maximum-likelihood from incomplete data via the EM algorithm. J. R. Stat. Soc. B.

[bib2] Dice L.R. (1945). Measures of the amount of ecologic association between species. Ecology.

[bib3] McLachlan G.J., Krishnan T. (1996). The EM algorithm and extensions.

[bib4] Rohlfing, T., Russakoff, D. B. & Maurer, C. R. 2003 Expectation maximization strategies for multi-atlas multi-label segmentation. In *Proc. Int. Conf. Information Processing in Medical Imaging*, pp. 210–221. 10.1007/978-3-540-45087-0_18 15344459

[bib5] Rohlfing T., Russakoff D.B., Maurer C.R. (2004). Performance-based classifier combination in atlas-based image segmentation using expectation-maximization parameter estimation. IEEE Trans. Med. Imag.

[bib6] Warfield, S. K., Zou, K. H. & Wells, W. M. 2002 Validation of image segmentation and expert quality with an expectation-maximization algorithm. In *MICCAI 2002: Fifth Int. Conf. Medical Image Computing and Computer-Assisted Intervention; 25–28 September 2002*, *Tokyo, Japan*, pp. 298–306. Heidelberg, Germany: Springer.

[bib7] Warfield S.K., Zou K.H., Wells W.M. (2004). Simultaneous truth and performance level estimation (STAPLE): an algorithm for the validation of image segmentation. IEEE Trans. Med. Imag.

[bib8] Warfield, S. K., Zou, K. H. & Wells, W. M. 2006 Validation of image segmentation by estimating rater bias and variance. In *MICCAI 2006: Ninth Int. Conf. Medical Image Computing and Computer Aided Intervention; 1*–*6 October 2006, Copenhagen, Denmark*, pp. 839–847. Heidelberg, Germany: Springer. 10.1007/11866763_103 17354851

